# A Township for the Blind

**Published:** 1915-02-20

**Authors:** 


### CENTRAL POOR-LAW CONFERENCE.

### A Township for the Blind.

THE most noticeable feature of the second day's proceedings of the Central Poor-Law Conference, last week, was the paper read by the Rev. G. A. Suttle, of the West Ham Board of Guardians, with reference to "The Poor-Law in Relation to the Blind and other Physical Defectives."

Mr. Suttle, in his paper, said that there were in England and Wales over 26,000 blind persons, of whom over 8,000 were assisted by Boards of Guardians. Quite 5,000 of these need not have been blind had they been properly attended to when they were born. The aged and infirm blind should be adequately pensioned without further ado; for to compel them to beggary or pauperism was an atrocity. But with regard to the younger and physically able a different course was advisable. They were credibly informed that there were in the United Kingdom about 12,000 persons capable of sustained and sustaining employment, and surely there could be nothing happier than that they be prepared for, and provided with, suitable employment. The presence of 6,000 blind persons in public places soliciting arms or inviting human pity by the exposure of their need was discreditable—not to them, but to the nation.

### The Bradford System.

The Guardians of Bradford had been specially commended in the House of Commons for the manner in which they discharged their duty to the blind. It might be summarised as follows: "Two Residential Training Homes are provided and managed by the Institution for the Blind, one being for men and the other for women. These Homes are almost wholly occupied by cases sent by the Guardians, and the Guardians pay the total cost of such cases, credit being taken for the value of the work done. When the training is completed the cases are transferred to the Central Institution. The Guardians do not, as a rule, contribute towards any cases employed at the Central Institution, on the ground that such an arrangement might have a tendency to encourage the other workers to look to the Guardians to supplement what they themselves earn, and thus encourage slackness. Out-relief is granted to suitable cases, and work is provided for these at their homes by the Institution. By arrangement with the police, blind beggars are given an opportunity of being trained in one of these Homes, otherwise they are dealt with by the magistrates. By this arrangement the streets are cleared of blind beggars, the Guardians do not have to provide accommodation for blind persons in the workhouse, and no such cases are in the workhouse unless they are suffering from some other disability. In addition to this, the Guardians have at times given lump sum subscriptions to the institution towards the deficit incurred by dealing with cases that would otherwise be chargeable to the Guardians."

### A City for the Blind.

The Bradford Union had struck a good line, at least so long as the nation considered that the semi-private provision for the blind was enough. When they were told that between two and three million pounds were invested in the interests of the blind and that something like £200,000 a year found its way into the coffers of these semi-private institutions, one felt proud of the donors and the dispensers of such charity. And yet one asked whether something better could not be devised, established, and sustained. Were he President of the Local Government Board he would seek and obtain powers to create a new township, directly under the authority of the Board, for the blind to dwell in. He would name it "Sunton," so that its very name should be a strict barrier to gloom. It should be laid out and cottaged as required, and have bakeries and other trading centres, together with proper municipal offices. He would establish workshops and factories for industries suitable for the blind, diverting such things as Government basket-making and other contracts from the private trader to his township. There should be adequate elementary, secondary and technical school accommodation. Music and other arts should be pursued. The manufacture of pianos should form part of his industrial scheme, as well as the tuning of them, thus relieving Germany of a little of her musical responsibility to the world. Experts should be there, not in too great numbers, to train and supervise.

Wages should be regulated by industry, with a minimum sufficient to insure decency and comfort. It might be asked whether he would limit the population of "Sunton" to the blind and the comparatively small number of sighted experts and teachers who would be directly engaged in its industries and schools. Such a restriction would be altogether unnecessary and undesirable. A certain proportion of sighted people would be required to perform municipal and other services outside the range of the blind, and quite a number of the capable blind were married and had families of sighted children who would amply contribute to the township's general liveliness. His idea was not the segregation of the blind, but the concentration of the capable blind for profitable co-operation with the advantage of direct and full interest in self-government, thus securing to them the best obtainable pleasures of responsible citizenship.

### The Discussion.

The Rev. H. S. Fullagar (Erpingham, Norfolk) said that Mr. Suttle's scheme was Utopian, and might not be workable. Was it intended that others besides the blind should be admitted to the township? The presence of officials was foreshadowed, and there would, of course, be other people as well. It was suggested that the blind in "Sunton" should follow certain trades by which they could support themselves. But there were many callings which the blind could not possibly follow, but which were necessary to the maintenance of any centre. He feared that the perfect ideal of happiness sketched by Mr. Suttle would not be found.

Mr. Charles Hughes, West Ham, said that the present method of dealing with the blind was unsatisfactory, and the problem was very acutely before the public now that officers and men were returning sightless from the war. The blind had a right to look to the public for assistance, but his experience showed him that they were exploited not so much by sweaters as by institutions masquerading under the guise of philanthropy. Great profits were made out of the blind, and his Board had withdrawn their patients from a blind institution on that very account. He did not like the idea of the blind having to apply for out-relief. Voluntary effort had failed in this as in so many other respects, and something else must be substituted. The State should take over the care of the blind, and provide for their education, training, and maintenance in circumstances tending to make them self-supporting.

Mr. J. Weller said he endorsed Mr. Suttle's suggestion that the Government should provide better opportunities for making the blind independent of charity in whatever form. The blind could do some work, such as boot-repairing, remarkably well. There was a great difficulty in dealing with blind musicians and hawkers, because their statements as to income could not be accepted. If he were a millionaire, he would give a bonus of 1s. to every 4s. received by blind people, and thus they would be able to ascertain the earnings of those who applied for help.

Mr. H. R. Farren (Coventry) said that the blind, together with certain other classes of physically defective persons, should be taken over by the State, and trained so that they should be, so far as possible, self-supporting.

Mr. John Hill (Northampton) said that from considerable experience he was satisfied that Mr. Suttle's scheme would not find favour with the blind. Institutions and funds for the blind were deriving an income of at least £200,000 a year from investments, but far too much of it was squandered on officials. There was too much delay and difficulty in getting a pension for a blind person; and, in short, voluntary effort should be superseded by the State, which should take over the whole of the funds now standing in the name of the blind. He proposed a resolution expressing satisfaction that the Government was seriously considering the problem of the blind, and urged the Government to make adequate provision for them at the earliest possible moment.

Mr. A. Chapman (Holborn) moved that it was "desirable as an experiment to form a township devoted to the benefit of blind persons who are chargeable to the State."

Mr. Hill's resolution was carried, but that of Mr. Chapman was defeated by an overwhelming majority.

